# The effects of flipped classroom versus lecture-based learning on academic performance and perceptions of classroom environment among nursing students: a randomized controlled trial

**DOI:** 10.1186/s12909-026-09746-y

**Published:** 2026-06-18

**Authors:** Mahboubeh Sadat Yousefi, Mohsen Fooladzadeh Dehghan, Mahnaz Ilkhani, Rahimeh Khajoei, Saeide Heidari, Mozhgan Jokar

**Affiliations:** 1 Department of Medical Surgical Nursing, School of Nursing and Midwifery, Qom University of Medical Sciences, Qom, Iran; 2 Department of Nursing, School of Nursing and Midwifery, North Khorasan University of Medical Sciences, Bojnurd, Iran; 3 Department of Medical Surgical Nursing, School of Nursing and Midwifery, Shahid Beheshti University of Medical Sciences, Tehran, Iran; 4 Department of Nursing and Midwifery, Sirjan School of Medical Sciences, Sirjan, Iran; 5 Nursing and Midwifery Care Research Center, School of Nursing and Midwifery, Isfahan University of Medical Sciences, Isfahan, Iran

**Keywords:** Academic performance, Classroom environment, Flipped classroom, Nursing students, Perception, Randomized controlled trial

## Abstract

**Introduction:**

Nursing students often struggle with the fluids and electrolytes course because of the complexity, extensive detail, and fleeting nature of the content. Evidence indicates that the flipped classroom approach can enhance learning and performance among students in the medical professions compared to traditional methods. This study aimed to compare the effects of two teaching approaches, the flipped classroom and traditional lecture-based learning, on nursing students’ academic performance and perceptions of the classroom environment.

**Methods:**

This was a single-blind, parallel-group, pretest-posttest randomized controlled trial. The study was conducted with 58 nursing students at Qom University of Medical Sciences, Iran, during the 2019–2020 academic year. Participants were randomly assigned using a computer-generated block randomization sequence to either the intervention group (flipped classroom, *n* = 29) or control group (lecture-based learning, *n* = 29). The primary outcome was academic performance, measured by researcher-developed 20-item multiple-choice knowledge test. The secondary outcome was perceptions of the classroom environment, assessed using the College and University Classroom Environment Inventory (CUCEI). Data were analyzed using descriptive and inferential statistics with SPSS version 26. All analyses followed the intention-to-treat principle and included all 58 randomized participants.

**Results:**

There was no statistically significant difference in the mean scores of knowledge test between the two groups prior to the intervention (t = 1.265, 95% CI: [-0.30, 1.33], *P* = 0.211). After controlling for pretest scores, ANCOVA revealed a significant and large effect of the flipped classroom on post-test knowledge (F = 104.719, *P* < 0.001, partial η² = 0.65). A one-way MANOVA on the seven CUCEI subscales yielded a significant multivariate effect (Pillai’s Trace = 0.93, F = 109.30, *P* < 0.001, partial η² = 0.93). With a Bonferroni-corrected alpha of 0.007, the flipped classroom group demonstrated significantly higher scores on Satisfaction, Involvement, Task Orientation, and Innovation, while no significant differences were found for Personalization, Student Cohesiveness, and Individualization.

**Conclusion:**

The findings suggest that the flipped classroom approach can improve nursing students’ academic performance and perceptions of the classroom environment. These results highlight the potential value of integrating active, student-centered pedagogies into nursing curricula. However, given the small sample size, short study duration, lack of long-term follow-up to assess knowledge retention, and single-institution design, further research with larger and more diverse samples is needed to confirm these results.

**Trial registration:**

The study was retrospectively registered in the Iranian Clinical Trials Registry (IRCT) with the trial number IRCT20240610062078N2 on December 21, 2025.

**Supplementary Information:**

The online version contains supplementary material available at https://doi.org/10.1186/s12909-026-09746-y.

## Introduction

The traditional lecture-based learning model has long been a cornerstone of nursing education, valued for its efficiency in delivering foundational knowledge to large cohorts of students. This approach ensures that all learners receive consistent, standardized content in a structured manner [1]. However, the breadth and complexity of contemporary nursing practice demand a more deliberate form of educational preparation that extends far beyond the simple transmission of information. Some scholars argue that exclusive reliance on lecture-based learning may not sufficiently equip nurses for rapidly evolving healthcare environments [2, 3]. Furthermore, existing educational approaches often fail to support students in applying critical thinking or transferring knowledge to new situations [4].

Lecture-based learning typically follows a one-way teaching approach in which the instructor takes an active role, attempting to deliver a large amount of information to students in a limited time [5]. While this method is cost-effective for presenting material to large groups, it is challenging to sustain students’ attention for more than 15 min [6]. In this approach, students often experience a disconnect between the theoretical knowledge they learn in class and the clinical skills they are expected to demonstrate at the bedside, reflecting a significant gap between nursing education and nursing practice [5, 7]. Without deliberate integration of interactive elements—such as questioning, case analysis, or brief problem-solving exercises— lectures rarely move beyond the remembering and understanding levels of Bloom’s taxonomy. This can limit opportunities for in-class application, analysis, and discussion, and reduces flexibility for one-on-one instructor-student interactions, potentially leaving struggling students behind [8–10]. The passive nature of this format can also present challenges in maintaining student engagement and can encourage a focus on rote memorization over deep learning [11].

Building on these concerns, there is a call for a fundamental transformation in nursing education [2]. Consequently, nursing education institutions worldwide have taken steps to redesign their educational approaches to address both the current demands of the healthcare system and students’ learning needs. In this context, educators are increasingly exploring student-centered pedagogical models designed to dedicate more in-class time to developing higher-order cognitive skills and better align academic training with modern educational practices [12, 13].

In response to the limitations of lecture-based learning, the flipped classroom has emerged as a prominent pedagogical approach for transforming passive educational settings into active learning environments [14, 15]. This approach, also known as the “inverted class”, was introduced in 2007 by Jonathan Bergmann and Aaron Sams, two chemistry teachers at Woodland Park High School in Colorado, United States. Their aim was to ensure that students who missed class for various reasons could keep pace with the curriculum and not be disadvantaged by their absence [8, 16]. This approach is deeply rooted in constructivist learning theory, which posits that learners actively construct their own knowledge through experiences and engagement [17].

From a pedagogical standpoint, the flipped classroom is a specific type of blended learning [18] that incorporates both online and face-to-face elements. In this approach, the learning environment shifts the educational environment from large group settings to individualized learning spaces and delivers course content outside the classroom. Instructors can create educational materials using modern technology formats such as videos, PowerPoint presentations, podcasts, notes, and animations, making them available to learners for access anytime and anywhere [2, 19]. In this approach, students acquire foundational knowledge through instructional videos prior to in-person sessions [20]. Class time is devoted to problem-solving, deep conceptual learning, group discussions, and the clinical application of existing knowledge [18, 21]. This approach allows students to build knowledge at their own pace and ensures that they arrive in class prepared to engage in discussions [7]. It also enables instructors to optimize their time and strengthen instructor-student interactions [22]. As a result, the lower levels of Bloom’s taxonomy—remembering and understanding—are addressed outside the classroom, while in-class time focuses on advancing higher-order cognitive skills (Applying, Analyzing, Evaluating, and Creating) [23, 24].

A growing body of evidence supports the flipped classroom in health professions education. In nursing specifically, flipped classroom has been associated with gains in knowledge, clinical skills, and learner satisfaction. For instance, Özkan et al. (2025) demonstrated that the flipped classroom positively influences nursing students’ knowledge and skill acquisition while also promoting long-term retention [17]. Kim et al. (2019) similarly reported significantly higher patient safety competency scores— in terms of knowledge, attitudes, and skills—among students taught via the flipped approach compared to those receiving lecture-based learning [25]. These findings are consistent with a recent meta-analysis confirming that flipped classroom enhances critical thinking, self-directed learning, and clinical competencies in nursing students [26]. These nursing-specific findings align with broader health professions evidence. A comprehensive systematic review by Naing et al. (2023), encompassing over 8,000 students across medicine, nursing, pharmacy, and allied health disciplines, concluded that the flipped classroom significantly improves academic performance compared to lecture-based learning [9]. Nevertheless, systematic reviews consistently note that the preponderance of high-quality flipped classroom studies in nursing education originates from the United States, Europe, and East Asia, with limited evidence from Middle Eastern educational contexts such as Iran [4, 27].

Iran’s nursing education system remains predominantly lecture‑driven, a pattern reinforced by large class sizes, limited faculty development in interactive methods, and uneven access to educational technology. While recent curricular directives encourage more student‑centered strategies, empirical evidence on the flipped classroom in Iranian undergraduate nursing programs is scant. A small number of studies have examined other courses [28, 29], but no published trial has focused on the Adult and Older Adults Nursing 1 course, which includes the notoriously challenging fluids and electrolytes module.

Within Iran’s nursing curriculum, the Adult and Older Adults Nursing 1 course, which covers topics such as fluids and electrolytes, gastrointestinal disorders, and orthopedics, is offered in the second semester of the bachelor’s nursing program. The fluids and electrolytes module is characterized by a high density of conceptual detail, and students across institutions consistently rank it among the most difficult topics in the curriculum [26, 27]. These difficulties are well documented in the nursing education literature, where the topic is frequently identified as one requiring innovative pedagogical approaches to enhance student comprehension and performance [30, 31]. At our institution, informal feedback gathered from end‑of‑course evaluations over several consecutive semesters repeatedly indicated that learners found the content abstract and difficult to retain. Notably, their scores on the fluids and electrolytes module were lower than those in other Adult and Older Adults Nursing topics, highlighting the need for targeted instructional interventions. Fluid and electrolyte imbalances are common in patients across their lifespan; nurses play a pivotal role in managing this balance [32]. Moreover, the life-threatening potential of these imbalances positions nurses as essential players in patient assessments and nursing care. Therefore, incorporating innovative educational approaches into nursing curricula to enhance students’ performance and learning in this challenging course is critically necessary. Accordingly, the present study was conducted to compare the effects of two teaching approaches, the flipped classroom and lecture-based learning, on the academic performance and perceptions of nursing students.

## Method

### Study design

In this study, a single-blind randomized controlled design with pre- and post-tests, including a control group, was employed to compare the effects of flipped classroom and traditional lecture-based learning on the academic performance and perceptions of nursing students. This study adhered to the Consolidated Standards of Reporting Trials (CONSORT) 2010 Statement, and the checklist is available in Supplemental File 1.

### Setting and participants

This study was conducted at the School of Nursing and Midwifery, Qom University of Medical Sciences, Iran, during the second semester of the 2019–2020 academic year. A total of 60 s-semester nursing students registered for the Adult and Older Adults Nursing 1 course and were initially assessed for eligibility. The inclusion criteria were as follows: taking the course for the first time, having access to devices such as mobile phones, computers, and the Internet for retrieving educational materials, and expressing willingness to participate in the study. Students were excluded if they had previously taken the course or were concurrently enrolled in another interventional educational study. After eligibility screening, two students were excluded because they had previously taken the course. Consequently, 58 eligible students were included in the study and underwent randomization.

### Randomization and blinding

After consent was obtained, the 58 participants were randomly assigned to the intervention group (*n* = 29) or the control group (*n* = 29). A blocked randomization sequence with randomly varying block sizes of 4 and 6 was generated by an independent statistician using the web-based service Sealed Envelope™ (https://www.sealedenvelope.com). This sequence ensured balanced group allocation throughout the enrollment period. To maintain allocation concealment, the generated sequence was implemented using sequentially numbered, opaque, sealed envelopes (SNOSE). A research assistant, who had no involvement in teaching, data collection, or data analysis, enrolled and assigned participants. This assistant provided each eligible student with the next sealed envelope in the sequence. The group assignment (intervention or control) was revealed to the participant only after the envelope was opened.

In the intervention group, teaching followed a flipped classroom approach, with pre-class materials provided for self-study and in-class time devoted to active learning activities. In the control group, instruction consisted of lecture-based learning delivered during class time with no required or assigned preparation beforehand. The instructor, classroom environment, class timing, and educational content were identical in both groups. To minimize the risk of contamination, students in the intervention group were explicitly instructed at the outset not to share pre-class materials or related discussions with peers in the control group.

This study employed a single-blind design. Due to the nature of the educational intervention, blinding of participants and the instructor was not feasible. However, all researchers involved in data collection and statistical analysis were blinded to group allocation. To ensure this, the lead investigator coded the groups as ‘A’ and ‘B’ in the dataset. The data analyst performed all analyses on this coded dataset and remained unaware of which group corresponded to the intervention until the analysis was complete.

### Sample size

The sample size was calculated based on the findings of a similar study [33], which indicated a significant difference in the mean score of knowledge post-test (primary outcome) between students participating in the flipped classroom (83.34 ± 9.81) and those receiving lecture-based learning (75.57 ± 9.82). According to the findings of the mentioned study and the following equation for mean comparison, the optimal sample size was estimated to be 25 students per group at a confidence interval of 95% and power of 80%. Considering a dropout rate of 10%, the required sample size was increased to 56 students (28 students in each group).

$$\:n=\frac{{\left({z}_{1-\frac{\alpha\:}{2}}+{z}_{1-\beta\:}\right)}^{2}\left({\delta\:}_{1}^{2}+{\delta\:}_{2}^{2}\right)}{{\left({\mu\:}_{1}-{\mu\:}_{2}\right)}^{2}}$$

### Study procedure and intervention design

#### Pre-intervention procedures

The intervention was implemented at the beginning of the academic semester to avoid any overlap with the mid-term or final exams. To facilitate course delivery, students were provided with a copy of the lesson plan approved by the Education Development Center (EDC), which encompassed the learning objectives, student assignments, required resources for instruction, teaching materials, assessment procedures, and a timetable for covering the course topics throughout the semester. During one session, the students were briefed on the study’s objectives and the mechanics of delivering instruction via the flipped classroom approach. At the beginning of the course, the instructor explicitly emphasized that completing the pre-class materials was essential for meaningful participation in subsequent in-class activities and for successfully engaging in collaborative problem-solving. They were also informed that exam scores would be collected exclusively for research purposes and would not factor in their final grades. Following this comprehensive briefing, students completed an informed consent form.

#### Course background

According to the lesson plan, the fluid and electrolytes topic was structured into four two-hour sessions delivered over four consecutive weeks. The session topics were:

- Session 1: Overview of the importance of water in the body and homeostatic mechanisms, along with an introduction to types of water imbalances (hypovolemia and hypervolemia)
- Session 2: Overview of electrolyte imbalances involving sodium ions (hyponatremia and hypernatremia) and potassium (hypokalemia and hyperkalemia)
- Session 3: Overview of electrolyte imbalances involving calcium ions (hypocalcemia and hypercalcemia) and magnesium (hypomagnesemia and hypermagnesemia)
- Session 4: Overview of electrolyte imbalances involving phosphorus ions (hypophosphatemia and hyperphosphatemia) and chloride (hypochloremia and hyperchloremia)

#### Interventions

The study comprised two parallel groups, as detailed below.

##### Intervention group: flipped classroom

Students assigned to the flipped classroom group attended four 2-hour sessions and were required to engage with pre-class materials made available online prior to each session. The in-class time was dedicated to interactive, collaborative learning activities. Under the guidance of the instructor, students worked in small groups to solve clinical case-based problems, participated in question-and-answer sessions, and engaged in discussions designed to apply the pre-class knowledge to practical scenarios. The instructor acted as a facilitator, providing targeted clarifications and fostering deeper understanding.

##### Control group: lecture-based learning

To ensure a valid comparison, participants in the control group attended four two-hour sessions covering the exact same learning objectives and core content as the intervention group. The sessions were delivered by the same instructor in a lecture-based learning format. Each session consisted of a didactic lecture using PowerPoint slides, followed by a period for student questions. The primary teaching activity was the direct presentation of course material by the instructor. To maintain educational equivalence, the PowerPoint slides used in the lectures were identical in content to those provided to the flipped classroom group. Students in the control group were given access to these slides for review after each session concluded.

#### Instructional materials

The instructional materials consisted of short topical video lectures and PowerPoint presentations, each lasting 10 to 15 min, supplemented by PDF handouts. All materials were developed by the primary instructor, who had over five years of teaching experience in fluids and electrolytes management. The materials were designed in accordance with Mayer’s Cognitive Theory of Multimedia Learning to optimize the integration of visual and auditory information. In addition, the overall content was structured based on cognitive load theory principles to ensure clarity and appropriate information chunking [34, 35]. Content validity was confirmed through peer review by six nursing education faculty members prior to implementation, ensuring accuracy, pedagogical soundness, and alignment with the course learning objectives.

#### Implementation of flipped classroom

##### Pre-class activities

To deliver instruction using the flipped classroom approach, the instructor provided the students with all the content intended for a single session one week in advance. In this preparatory phase, students were required to review the educational materials at home or in a non-classroom setting and arrive prepared for the subsequent session. The materials distributed included short topical videos and PowerPoint presentations (each 10–15 min long), along with a PDF of the lecture notes, all uploaded to the virtual educational group hosted on Telegram. In our setting, Telegram was already the primary communication tool used by students for academic purposes, and all participants had it installed and used it regularly. Choosing Telegram therefore minimized access barriers, allowed students to receive notifications and materials on their smartphones in real time, and enabled rapid two-way communication within the class group. At the end of each video and presentation, a question or brief scenario was included to guide self-directed learning and gauge students’ understanding of the intended learning outcomes, thereby prompting students to pause, take notes, and actively process the content rather than merely watching it passively. Students were encouraged to review these questions and bring their reflections to class to support discussion and group work. However, the completion or submission of these preparatory tasks was not formally recorded or verified during this study; instead, we relied on classroom observations and the quality of students’ contributions during in-class discussions as indirect indicators of their pre-class engagement. This issue is particularly important because pre-class preparation time is a key practical concern in health professions education, where student workloads are high. To estimate preparation time, students were asked to complete a brief, weekly self-reported log indicating the approximate time they spent on pre-class materials. These logs, collected via a simple online form, indicated that these tasks required approximately 60–80 min per session on average (50–60 min for viewing the videos plus approximately 20 min for answering the questions).

Additionally, for in-class sessions, the instructor prepared questions and clinical scenarios aligned with each session’s core concepts for use during class.

##### In-class activities

1. At the start of the class, the instructor spent 10–15 min addressing students’ questions and clarifying misunderstandings about the educational content in order to resolve any misconceptions.
2. The instructor engaged individually with the students regarding the questions raised in the pre-class materials, which took approximately 15 min.
3. The students were divided into groups of five to six members to discuss the scenarios and questions prepared by the instructor. This involved the instructor presenting predesigned clinical scenarios using PowerPoint. Each group was given a few minutes to deliberate and collaborate internally in order to formulate their responses. A representative of the group presented the answer in this scenario. Members of other groups could offer comments and ask questions. Finally, the instructor addressed any remaining issues and student queries. In this way, students practically applied their theoretical knowledge by analyzing and responding to clinical scenario questions. This phase lasted for approximately 70–90 min.

### Data collection tools

Three tools were used in this study: the demographic questionnaire, fluid and electrolyte knowledge tests, and the College and University Classroom Environment Inventory (CUCEI).

1. The demographic and educational questionnaire included age, gender, marital status, and overall grade point average (GPA), which were completed by students via self-reporting.
2. To assess academic performance (primary outcome), pre- and post-intervention test scores were used to measure the students’ knowledge gains. The tests consisted of 20 multiple-choice questions (with four options each) on knowledge related to fluid and electrolyte topics. Each correct answer was worth 1 point, with scores ranging from 0 to 20; higher scores indicated better knowledge of fluids and electrolytes.

The pre- and post-test questions were identical and designed by two members of the research team (not the lecturer) with several years of experience teaching the fluid and electrolytes course; the questions were based on the learning objectives (See Supplementary File 2).

After designing the knowledge questionnaire, its validity and reliability were assessed. Content and face validity were established through both qualitative and quantitative expert reviews. A panel comprising nursing faculty and clinical specialists in nursing and internal medicine evaluated the qualitative validity of the items, and the instrument was refined through iterative revisions based on their consensus. For quantitative validation, the content validity index (CVI) was calculated. The same 10 experts rated the relevance of each item on a 4-point scale ranging from 1 (not relevant) to 4 (highly relevant), yielding a CVI of 0.98, which confirmed satisfactory content validity. Internal consistency reliability was assessed using the Kuder‑Richardson Formula 20 (KR‑20), the appropriate index for dichotomously scored items (correct/incorrect). The obtained KR‑20 value of 0.84 indicated good internal consistency.

Item analysis confirmed that both the difficulty and discrimination indices of the test questions were optimal. The mean difficulty index was 52%, which falls within the ideal range of 30–70%. The mean discrimination index was 0.31, exceeding the threshold for good item discrimination (≥ 0.30).

To reduce potential practice effects arising from repeated exposure to the same knowledge test, the order of both the items and the answer options within each item was altered in the post test. This procedure minimizes recognition-driven responses based on item-position memory while preserving content equivalence.

3. The CUCEI was used to assess students’ perceptions of the classroom environment (secondary outcome), which was developed and validated by Fraser, Treagust, and Dennis (1986) [36]. This tool is specifically designed for higher education settings and can be administered in either actual or preferred versions. In the actual version, students reported their observations and opinions about the class they were currently attending, while the preferred version captured their expectations and preferences for an ideal classroom. This study employed the actual version, which consists of 49 items across 7 scales (7 items per scale):
  Personalization (emphasis on opportunities for individual student-instructor interactions)
  Involvement (degree of active and attentive student participation in class discussions and activities)
  Student Cohesiveness (extent to which students know, help, and are friendly towards each other)
  Student Satisfaction (level of enjoyment that students derive from the class)
  Task Orientation (degree of clear organization and structure in class activities)
  Innovation (instructor’s planning of novel and unconventional class programs and activities)
  Individualization (students’ autonomy in decision-making and treatment based on their individual differences and abilities)

The questionnaire items were rated on a Likert scale with options of strongly agree, agree, disagree, and strongly disagree. Approximately half of the items required reverse-scoring. For positively worded items, the scoring was as follows: strongly agree = 5 points; agree = 4 points; disagree = 2 points; and strongly disagree = 1 point. For negatively worded items: strongly agree = 1 point; agree = 2 points; disagree = 4 points; and strongly disagree = 5 points. Unanswered items or those marked as “no opinion” received three points, regardless of item wording. Each scale yielded a score ranging from 7 to 35, with a total questionnaire score ranging from 49 to 245. Higher scores indicated more positive student perceptions of the learning environment, either overall or on an individual scale. The internal consistency (Cronbach’s alpha) ranged from 0.7 to 0.9 across the seven scales [36].

The Persian version of the CUCEI questionnaire has been validated in Mohammadi’s study and used in many other studies [37, 38]. Cronbach’s alpha for the seven subscales in the present sample ranged from 0.73 (Task Orientation) to 0.92 (Involvement), indicating acceptable to good internal consistency. The overall scale alpha was 0.92.

Research assistants collected data at two time points over four weeks. One week prior to the in-person classes and exposure to the course content, a pre-test was administered to the control and experimental groups. At the end of the fourth session and upon completion of the intervention, the CUCEI and post-test were completed by the students in the two groups. As students could not be blinded to their teaching method, self‑report responses on the CUCEI are susceptible to response bias (e.g., social desirability, halo effect). To mitigate this, the data collection was conducted by neutral research assistants, and students were assured that responses were anonymous and unrelated to course grades.

### Data analysis

Data were analyzed using SPSS version 26. The normality of the data was assessed using the Shapiro-Wilk test. Descriptive statistics are presented as mean and standard deviation for continuous variables, and as frequency and percentage for categorical variables.

The baseline comparability of the two groups on categorical demographic variables was assessed using the chi‑square test; Fisher’s exact test was applied when any expected cell frequency was below five.

Pre‑test knowledge scores were compared between the intervention and control groups using an independent‑samples t‑test, with the assumptions of normality (Shapiro–Wilk: control, *P* = 0.103; intervention, *P* = 0.058) and homogeneity of variances (Levene’s test: *P* = 0.06) confirmed as tenable.

For the primary outcome, between‑group differences in post‑test knowledge scores were examined using a one‑way analysis of covariance (ANCOVA), with the pre‑test score entered as the covariate. All ANCOVA assumptions—normality of residuals, homogeneity of regression slopes, and homogeneity of variances—were examined and found to be satisfied. Effect size was reported as partial eta squared (partial η²).

Within‑group changes from pretest to posttest were assessed to describe the magnitude of improvement within each arm. The paired‑samples t‑test was used for the control group, as the difference scores met the normality assumption (Shapiro–Wilk, *P* = 0.098). For the intervention group, the Wilcoxon signed‑rank test was applied because the difference scores violated normality (Shapiro–Wilk, *P* = 0.025). The 95% confidence interval for Hodges–Lehmann pseudo‑median is reported for the non‑parametric comparison.

To compare students’ perceptions of the classroom environment across the seven subscales of the CUCEI, a one‑way multivariate analysis of variance (MANOVA) was conducted. This omnibus test controls the familywise error rate when multiple correlated outcomes are examined simultaneously. Effect sizes for the MANOVA and each follow‑up ANOVA are reported as partial η².

The primary analysis was conducted according to the intention‑to‑treat (ITT) principle; all participants who underwent randomization were included in the analysis in their originally assigned groups, regardless of any protocol deviations. A significance level of *P* < 0.05 was adopted for all inferential tests unless otherwise corrected.

## Results

**Fig. 1:**
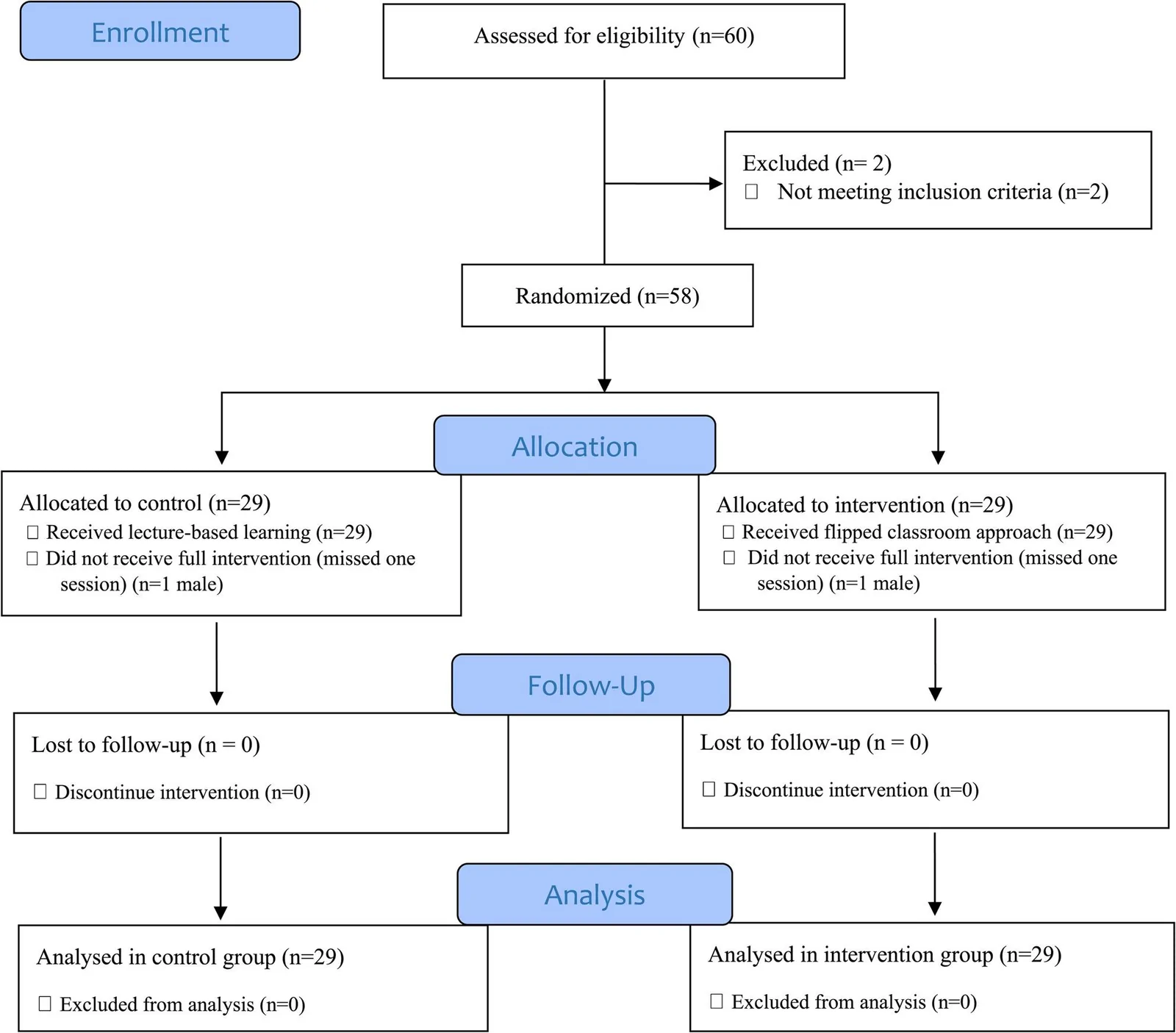
Flow diagram of the study

Figure 1 presents the trial diagram illustrating participant flow through the study. Protocol deviations, predefined as missing one or more of the four scheduled educational sessions or failing to complete the study questionnaires, occurred in two cases; one participant in each group missed a single session. Importantly, in both instances, the absence occurred during a non‑final session and did not coincide with the administration of post‑test outcome measures. Consequently, complete post‑test data were obtained from all 58 randomized participants; there was no loss to follow‑up and no missing outcome data. In accordance with the ITT principle, all 58 participants were retained in their originally assigned groups for primary analysis.

The overall mean age of the students was 20.86 ± 2.96 years, with the majority being single (89.7%); their overall GPA was 16.02 ± 1.61. As shown in Table 1, the results showed no statistically significant differences in students’ mean age, GPA, and marital status between the intervention and control groups (*p* ˃ 0.05).

**Table 1:** Sociodemographic and educational characteristics of participants in the flipped classroom and lecture-based learning groups

	Lecture-based learning (*n* = 29)	Flipped classroom (*n* = 29)	Total (*n* = 58)	Test statistic	*P* value
Age	20.82 ± 3.41	20.89 ± 2.49	20.86 ± 2.96	t = 0.088	0.930^a^
Sex					
Male	12 (41.4)	16 (55.2)	28 (48.3)	χ² = 1.105	0.293^b^
Female	17 (58.6)	13 (44.8)	30 (51.7)		
Marital status					
Single	27 (93.1)	25 (86.2)	52 (89.7)	χ² = 0.744	0.670^c^
Married	2 (6.9)	4 (13.8)	6 (10.3)		
GPA	15.73 ± 1.68	16.30 ± 1.52	16.02 ± 1.61	t = 1.347	0.183^a^

Data are presented as mean ± SD for continuous variables and as n (%) for categorical variables

^a^ Independent samples t-test

^b^ Chi-square test (applied when all expected cell frequencies ˃ 5)

^c^ Fisher’s exact test (applied when any expected cell frequency ˂ 5)

The results of the independent-samples t-test showed that there was no statistically significant difference between the mean scores of the knowledge test before the intervention in the two groups (t = 1.265, 95% CI: [–0.30, 1.33], *P* = 0.211). Consequently, ANCOVA was conducted on the post‑test scores, controlling for the pre‑test as the covariate, in order to adjust for any baseline variation and obtain a purer estimate of the intervention effect.

The ANCOVA results, displayed in Table 2, revealed a statistically significant difference in adjusted post‑test means between the intervention and control groups (F = 104.719, *P* < 0.001). The effect size was large (partial η² = 0.65), indicating that 65% of the variance in post‑test scores could be attributed to the teaching method rather than to chance. In conclusion, these findings highlight the substantial practical benefits of flipped classroom approach in boosting students’ knowledge test scores.

**Table 2:** Descriptive statistics and ANCOVA results for knowledge test scores in flipped classroom and lecture-based learning groups

Group	Pre test mean ± SD	Post test mean ± SD	ANCOVA for between-group comparison					
			Adjusted Post-test Mean (SE)	Mean Difference^a^	95% CI for Difference^a^	F	P value	Partial. η2
Lecture-based learning (*n* = 29)	5.58 ± 1.78	12.24 ± 2.19	12.49 (0.24)	3.59	(2.89, 4.30)	104.719	< 0.001	0.65
Flipped classroom (*n* = 29)	6.10 ± 1.29	16.34 ± 1.79	16.09 (0.24)					

*SE* Standard Error

^a^ Mean difference and 95% CI reflect the adjusted post-test scores of the intervention group minus the control group. Positive values indicate higher scores in the intervention group

Within‑group analyses further indicated that knowledge scores improved significantly from pre-to post-test in both groups. Change scores were calculated as post-test minus pre-test. In the control group, the paired‑samples t‑test revealed a significant increase (t = 26.673, 95% CI: [6.14, 7.16], *P* < 0.001). In the intervention group, the Wilcoxon signed‑rank test similarly confirmed a significant improvement (Z = 4.742, 95% CI for the Hodges–Lehmann pseudo‑median: [10.00, 10.50], *P* < 0.001). While both groups improved, the between‑group ANCOVA results demonstrated that the magnitude of improvement was significantly greater in the flipped classroom condition.

A one-way MANOVA was conducted to examine the effect of teaching method on the seven subscales of the CUCEI. The multivariate effect was statistically significant, Pillai’s Trace = 0.93, F = 109.30, *p* < 0.001, partial eta squared = 0.93, indicating a very large overall effect. Follow-up univariate ANOVAs were performed on each subscale. A Bonferroni correction was applied to control for multiple comparisons, yielding an adjusted alpha level of 0.007 (0.05 / 7). Based on this corrected criterion, the flipped classroom group scored significantly higher than the lecture-based learning group on four of the seven subscales: Satisfaction, Involvement, Task Orientation, and Innovation. The remaining three subscales—Personalization, Cohesiveness, and Individualization—did not reach statistical significance after the correction. All descriptive and inferential statistics are reported in Table 3.

**Table 3:** Comparison of CUCEI subscale scores between flipped classroom and lecture-based groups: univariate follow-up analyses following significant MANOVA

	Lecture-based learning		Flipped classroom		95% CI	F	*P* value^a^	Partial Eta Squared
Subscale	Mean	SD	Mean	SD				
Personalization	21.37	3.73	24.44	5.55	(0.57, 5.56)	6.08	0.017	0.098
Involvement	16.75	3.34	30.72	1.92	(12.53, 15.40)	379.688	< 0.001	0.871
Student Cohesiveness	21.89	5.87	22.17	4.40	(-2.45, 3.00)	0.041	0.840	0.001
Satisfaction	19.58	3.33	29.37	3.06	(8.12, 11.46)	137.621	< 0.001	0.711
Task Orientation	20.62	1.71	28.72	2.54	(6.96, 9.24)	201.499	< 0.001	0.783
Innovation	15.89	2.31	29.48	2.33	(12.36, 14.81)	493.148	< 0.001	0.898
Individualization	17.41	5.79	16.31	5.81	(-4.15, 1.95)	0.524	0.472	0.009

^a^ To account for multiple comparisons across the seven CUCEI subscales, a Bonferroni-adjusted alpha level of 0.007 (0.05 / 7) was used. Statistical significance was interpreted based on this corrected criterion

## Discussion

This study aimed to compare the effects of the flipped classroom approach and the lecture-based learning on the academic performance and classroom environment perceptions of nursing students. The primary finding of this study was that the flipped classroom approach led to a significant improvement in nursing students’ academic performance in the fluids and electrolytes course compared to lecture-based learning. This result aligns with a substantial body of evidence suggesting that active learning pedagogies are more effective than passive instruction in nursing education. For example, studies by Mengesha et al. (2024) and Joseph et al. (2021) similarly found higher exam scores in courses such as pharmacology, anatomy and physiology after implementing a flipped approach [2, 39]. The convergence of these findings suggests that the benefit is not topic-specific but stems from the core principles of the flipped classroom approach. By requiring students to engage with foundational knowledge before class, in-person sessions—much like in our study—could be dedicated to higher-order problem-solving and application, which likely solidifies understanding and leads to better performance on assessments. This interpretation is further strengthened by a recent meta-analysis that confirmed that flipped learning provides a significant advantage for theoretical knowledge acquisition among nursing students [40].

While our study focused on academic performance and classroom perceptions, it is worth considering how the positive environment we documented might relate to the development of professional skills reported in the literature. It is plausible that such an engaging and goal-oriented environment can create conditions conducive to fostering professional attributes. Specifically, prior studies have documented improvements in cardiopulmonary resuscitation [41], urinary catheterization [42], safe medication administration [43], and physical examination skills when instruction was delivered using pre-class learning and active in-class application [44]. In addition, emphasis on in-class problem-solving is consistent with pedagogies aimed at enhancing critical thinking [45, 46]. Similarly, a collaborative atmosphere may support the development of soft skills, such as teamwork and communication [47]. However, our study did not measure any clinical, professional, or soft skills. Therefore, whether the enhanced learning environment observed in our study translates into measurable improvements in these broader competencies is a hypothesis that requires direct investigation in future studies.

However, it is important to contextualize our findings within a literature that includes conflicting results. For instance, Sourg et al. (2023) found no significant academic advantage for a flipped classroom in their study of medical students [48]. An analysis of their methodology revealed key design differences that likely explain this discrepancy. First, their control group received reading materials immediately before the post-test, an intervention that deviates from standard lecture protocols and may have artificially enhanced their performance. Second, the students in their study were given a very short preparation time for a pedagogical approach that was new to them. Inadequate preparation time is a known barrier to the effectiveness of the flipped approach and offers a more direct explanation for their null findings compared to the positive results observed in our study.

It is evident that several factors influence the effectiveness of flipped classroom experiences. One key factor is ease of access and unlimited availability of videos and educational resources. Students have the flexibility to repeatedly review videos and materials at a time and place that suits them best, fostering a deeper understanding of the content and allowing them to learn at their own pace. Additionally, pre-class activities boost students’ curiosity and motivation while highlighting potential misconceptions about the topic [49]. Consequently, when students enter in-person sessions equipped with foundational knowledge gained through pre-class learning activities [12], instructors facilitate the application of this knowledge to clinical contexts by designing in-class clinical scenarios. This exposure enables nursing students to engage in the practical use of their prior knowledge and develop higher-order thinking skills. Moreover, instructors gain more time and opportunity to provide feedback to students and correct any misconceptions from the pre-class materials. Overall, pre-class learning, content recall, and accurate conceptual understanding all contribute to elevating students’ learning outcomes in the flipped classroom.

The present study further showed that students perceived the flipped classroom environment as substantially more positive than the lecture-based environment across several subscales of the CUCEI. Students exposed to the flipped classroom reported significantly higher levels of involvement, satisfaction, task orientation, and innovation. The most pronounced advantages emerged in the domains of involvement and innovation, suggesting that students perceive flipped classrooms as markedly more creative, active, varied, and novel in teaching methods than lecture-based learning. This perception is not surprising, given that flipped designs explicitly incorporate diverse active-learning techniques—peer instruction, case-based discussions, and technology-enhanced activities—whereas lectures remain predominantly instructor-centered and didactic.

The overwhelmingly positive student perceptions recorded in our study, particularly the high scores for classroom ‘Involvement’ and ‘Innovation’, are consistent with a broader trend in nursing education [33]. Our results suggest that this positive view is not arbitrary but is rooted in specific, valued pedagogical mechanisms. The enthusiasm our students showed for a more active and engaging environment is directly explained by research from El Sadik and Al Abdulmonem (2021), who found that students’ positive attitudes were driven by meaningful pre-class activities that led to richer peer and instructor interactions [50]. This interactive component appears to be a key driver of cognitive engagement. For example, Ng EKL (2023) identified peer interaction and the opportunity to apply knowledge as the primary reasons for improved student engagement and better exam performance [51]. This shift towards active, self-directed learning is also highly valued by students, as shown in a survey by Al Suliman et al. (2025), in which over 70% of students confirmed that the flipped approach improved their efficiency and fostered self-directed learning [15]. Taken together, these elements—engaging activities, meaningful interaction, and student autonomy—naturally culminate in a more positive overall experience, a conclusion supported by a meta-analysis showing that the flipped classroom consistently enhances nursing students’ learning satisfaction [26].

However, while these perceptual differences are substantial, they must be interpreted with caution, as several factors beyond the pedagogical method itself could have contributed to these outcomes. First, the introduction of a new teaching method may have triggered a ‘novelty effect,’ where the newness and change of pace temporarily boosted students engagement and satisfaction, irrespective of the method’s long-term efficacy. Second, a potential ‘instructor effect’ cannot be ruled out; the educator’s own enthusiasm and heightened preparation for implementing a new intervention could have inadvertently influenced the student’s perceptions. More importantly, the most significant limitation affecting the CUCEI results is the non-blinded nature of the study. Participants were fully aware of their group allocation, creating a high risk of response bias. Students in the experimental group, knowing that they were receiving an innovative intervention, may have felt inclined to report more positive experiences to please the instructor or to align with perceived expectations of the study—a phenomenon known as social desirability bias or the Hawthorne effect. This bias is particularly problematic for subjective self-report measures, such as the CUCEI. Consequently, it is plausible that the large effect sizes observed for subscales such as Involvement and Satisfaction are partially inflated by this methodological artifact. Therefore, while the CUCEI results strongly suggest a positive student experience, they should be interpreted as indicative of the direction of the effect rather than a precise measure of its magnitude.

Notably, no significant differences emerged on the Student Cohesiveness, Individualization and Personalization scales, a finding that warrants careful interpretation. Regarding Student Cohesiveness, which measures peer support and friendship within the class, our results suggest that simply incorporating group work does not automatically foster a greater sense of community. While our flipped classroom included collaborative activities, these were primarily short, task-focused exercises rather than long-term projects designed to build deep interpersonal bonds. It is plausible that for cohesiveness to develop, collaborative tasks must be intentionally structured to promote social connections, not just content mastery. Furthermore, the duration of the intervention may have been insufficient for the formation of strong peer relationships.

The lack of difference in Individualization is also informative. This scale assesses the degree to which students feel the instructor caters to their personal needs and learning pace. One might have expected the flipped approach to increase this perception, as instructors are theoretically freed up for more personalized interactions. However, our findings suggest that this freed-up time in the classroom was likely redirected from individual teacher-student interactions to the facilitation of group discussions. Therefore, while the pedagogical method changed, the perceived level of individualized attention from the instructor remained the same. This highlights a critical insight: the benefits of the flipped classroom are not universal across all environmental metrics and depend heavily on the specific design and focus of in-class activities.

The absence of a significant difference in Personalization is particularly noteworthy. In flipped classroom approaches, proponents often argue that by shifting content delivery to pre-class activities, instructors gain more in-class time for one-on-one interactions and individualized feedback—conditions that should theoretically enhance perceived personalization. Yet the present findings suggest that structural changes alone do not guarantee this outcome. One plausible explanation is that merely reallocating class time does not ensure that instructors utilize it in ways students experience as personally responsive. If the additional face-to-face time is devoted to standardized group activities rather than differentiated, learner-specific interactions, the subjective experience of personalization may remain unchanged from that of a traditional lecture setting. Furthermore, even a moderately sized class, while typical, can constrain the frequency and depth of individual student–instructor interactions, limiting the extent to which students feel personally recognized. This finding highlights the importance of equipping instructors with concrete strategies—such as individualized feedback on pre-class work, deliberate use of student names, and one-on-one progress check-ins—to maximize personalization when adopting flipped designs, particularly in classes where group-based activities dominate in-class time.

In contrast to our findings, Sajid et al. (2020) reported largely negative student perceptions of the flipped classroom. This resistance is not uncommon and is often linked in the literature to specific challenges inherent in this pedagogical shift [52]. Specifically, the increased workload from pre-class activities is frequently cited by students as a primary source of dissatisfaction, as they may perceive it as an additional burden rather than an integral part of the learning process. Therefore, the negative outlook reported by Sajid et al. is likely a direct reflection of these well-documented implementation challenges, highlighting the need for careful student preparation to ensure the model’s success.

### Strengths and limitations

A key strength of this study is its use of a randomized controlled trial to rigorously evaluate the flipped classroom for teaching a specific, critical topic such as fluids and electrolytes to nursing students within the Iranian context. However, the findings must be interpreted in light of several significant limitations.

The limitations of this study include the small sample size and the fact that only four sessions of the two-credit nursing course were delivered in flipped format. This short timeframe has important consequences: it may be insufficient for the initial “novelty effect” to wear off, and it makes it difficult to assess the sustainability of the pedagogical impact over time. Given that students were encountering this approach for the first time in their education, it should be extended across more sessions throughout the semester in order to identify its strengths and weaknesses more clearly. Relatedly, our study did not assess the long-term retention of knowledge, as the design did not include a delayed post-test. Therefore, we cannot draw conclusions about the lasting impact of the flipped classroom on memory, and future studies are needed to investigate this outcome. Furthermore, the study was conducted at a single nursing school, which may limit the generalizability of our findings to other academic and cultural contexts.

Another potential limitation of this study is the risk of contamination between groups, as students in the intervention group could have shared pre-class materials with those in the control group through informal interactions. No formal structural measures were implemented to prevent interaction between groups, as all participants were enrolled in the same academic program and semester, which naturally allowed for informal communication outside of class. However, at the start of the intervention, students in the flipped classroom group were explicitly instructed not to share pre-class materials or discussions with peers in the control group.

Several methodological issues also introduce potential biases that warrant caution. A critical limitation is the lack of blinding for both participants and the instructor. From the participants’ perspective, awareness of being in the intervention group could have triggered a novelty effect or a Hawthorne effect, inflating their self-reported perceptions on the CUCEI due to social desirability bias. It is recommend that future studies include longer follow‑up periods to determine whether the gains persist beyond the initial exposure to a new instructional format. From the instructor’s perspective, knowledge of the group assignments prevents us from disentangling the effectiveness of the pedagogy from the ‘instructor effect,’ as unintentional variations in enthusiasm or support for the novel method could have influenced outcomes. Another methodological limitation was the use of an identical assessment for both the pre-test and post-test. This design creates a risk of a “testing effect,” where exposure to the test questions during the pre-test may have contributed to students’ improved scores on the post-test, irrespective of the pedagogical intervention. Simply put, familiarity with the question format and content could account for a portion of the observed learning gain. To mitigate this bias in future research, the use of parallel or alternate forms of the assessment is highly recommended.

Finally, several unmeasured factors may have acted as confounding variables. The first is the absence of formal tracking of students’ engagement with the pre-class materials. Although students in the flipped condition were encouraged to complete preparatory activities and answer related questions, their actual level of participation was not systematically monitored. This unmeasured variability in preparation may have influenced the learning outcomes. Future research should therefore incorporate structured tracking mechanisms, such as pre-class quizzes, to better capture the extent and quality of pre-class engagement in the flipped classroom. Lastly, the use of the Telegram application for communication exclusively within the intervention group introduced another potential confounder, as no equivalent channel was provided for the control group. Future research should aim to address these limitations through multi-center designs, longer interventions, objective tracking of student preparation, and robust assessment strategies.

## Conclusion

In this single-center controlled trial, the flipped classroom approach resulted in significantly improved immediate post-test knowledge scores and more positive student perceptions of the learning environment in a fluids and electrolytes course. However, these encouraging findings must be interpreted with considerable caution in light of the study’s methodological limitations. The results are confined to short-term knowledge gains, as long-term retention was not assessed. Furthermore, the findings’ generalizability is constrained by the single-institution design, small sample size, and short intervention duration. The non-blinded nature of the study also means that the highly positive perceptual outcomes should be viewed as preliminary. Therefore, while the flipped classroom appears to be a promising pedagogical strategy within this specific context, its broader effectiveness and sustainability in nursing education cannot be confirmed without further, more rigorous research that includes multi-center designs, larger sample sizes, and long-term follow-up.

## Supplementary Information


Supplementary Material 1.



Supplementary Material 2.


## Data Availability

The datasets analyzed during the current study are not publicly available due to ethical considerations, but are available from the corresponding author on reasonable request.
