# Supplementary material for: The effects of flipped classroom versus lecture-based learning on academic performance and perceptions of classroom environment among nursing students: a randomized controlled trial
Source: BMC Med Educ. 2026 Jun 18;26:1396. doi: 10.1186/s12909-026-09746-y (PMC13523480; doi:10.1186/s12909-026-09746-y)
Supplement: Supplementary file 1 [file 12909_2026_9746_MOESM2_ESM.docx]

**Test your knowledge**

1. In which of the following individuals is the total body water percentage typically the highest?

A) 19-year-old man

B) 19-year-old woman

C) 50-year-old man

D) 50-year-old woman

2. The nurse is caring for a client who is taking a diuretic to treat heart failure. Which of the following signs is more likely to be present in the patient?

A) Pulmonary rales

B) High blood pressure

C) Decreased hematocrit

D) Decreased CVP

3. A 30-year-old man with pyloric stenosis presented with recurrent vomiting. What IV fluids do you expect the physician to prescribe for this patient?

A) Ringer's lactate

B) Normal saline

C) 5% dextrose

D) 10% dextrose

4. In large infusions of intravenous fluids that do not contain electrolytes, such as 5% dextrose, the likelihood of disturbance in which of the following electrolytes is highest?

A) Potassium

B) Sodium

C) Calcium

D) Phosphorus

5. While checking a client’s morning laboratory report, the nurse notes a BUN level of 60 mg/dL. Which clinical findings is NOT consistent with a high BUN level?

A) Kidney failure

B) Liver failure

C) GI bleeding

D) Dehydration

6. Upon the patient's arrival at the emergency department, after reviewing their lab report, the doctor prescribed a salbutamol inhaler. Given that the patient has no signs of respiratory issues, what do you think is the reason?

A) Hyperkalemia

B) Hypernatremia

C) Hypercalcemia

D) Hypermagnesemia

7. Which of the following electrolyte disorders can cause digoxin toxicity?

A) Na 150 mEq/L

B) Ca 7.8 mg/dL

C) K 2.9 mEq/L

D) Mg 3 mg/dL

8. An elderly patient developed fatigue, weakness, muscle cramps, and cardiac arrhythmia after receiving laxatives for colonoscopy preparation. The most likely electrolyte abnormality was:

A) Hypocalcemia

B) Hypomagnesemia

C) Hypokalemia

D) Hyponatremia

9. Which patient below would have a sodium level of 124 mEq/L?

A) SIADH

B) Watery diarrhea

C) Renal failure

D) Insufficient fluid intake

10. A 75-year-old patient with diabetes was brought to the emergency department with decreased consciousness. Initial tests are as follows: Cr=2.5 mg/dL, BUN=28 mg/dL, Na=147 mEq/L, BS=800 mg/dL. The patient was diagnosed with diabetic ketoacidosis. The nurse initiates treatment with patient hydration and intravenous insulin as per the orders. With the initiation of insulin and fluid therapy, which of the following electrolyte disorders is possible?

A) Hyponatremia and hypokalemia

B) Hypokalemia and hypomagnesemia

C) Hypokalemia and hypocalcemia

D) Hypomagnesemia and hyponatremia

11. If a patient's total serum calcium level is 10 mg/dL and serum albumin level is 2.5 g/dL, what is the corrected calcium level in mg/dL?

A) 11.2

B) 8.8

C) 12.5

D) 11.5

12. The nurse is caring for a client with a serum potassium level of 7 mg/dl. Which of the following electrocardiogram changes would the nurse expect to observe?

A) Loss of P-wave

B) Wide QRS

C) U-wave

D) Tall T wave

13. A nurse manually measures a patient’s blood pressure. As she pumped up the cuff above the systolic pressure for a few minutes, she noticed that the patient developed a carpal spasm. The nurse knows that the client has which of the following conditions?

A) Hypercalcemia

B) Hypocalcemia

C) Hyperkalemia

D) Hypokalemia

14. The nurse is reviewing the lab values of a client and notes a serum calcium level of 7 mg/dl. The nurse knows that the client may be at high risk for which of the following conditions, except:

A) Insufficient vitamin D intake

B) Kidney failure

C) PH=7.8

D) Immobility

15. The nurse is caring for a client who was previously alert, oriented, and ambulatory. The nurse notes that this client can no longer lift his arms and is beginning to demonstrate erratic behavior, such as attempting to climb out of bed. Laboratory analysis revealed a sodium level of 125 mEq/L. Which of the following medications is the nurse likely to administer?

A) Hydrochlorothiazide

B) Indapamide

C) Mannitol

D) Haloperidol

16. A 61-year-old client was seen at a healthcare clinic for help with the management of kidney disease. The patient was in renal failure and had high phosphorus levels. Which foods should the nurse counsel the client to avoid in this situation?

A) Vegetables

B) Chicken

C) Egg whites

D) Olive oil

17. A nurse is caring for a client and notes confusion, numbness, muscle twitching and tingling, and abdominal distention. The nurse suspected hypomagnesemia. Which question by the nurse would be most appropriate to determine a possible cause for this condition?

"Do you smoke cigarettes every day?"

"Do you drink alcohol every day?"

"Do you drink milk every day?"

"Do you take recreational drugs every day?"

18. A nurse is reviewing the lab values of a client and notes a serum chloride level of 110 mEq/L. Which patient is at risk for this condition?

A) A 25-year-old woman with cystic fibrosis.

B) A 55-year-old postoperative patient from abdominal surgery with a nasogastric tube with continuous suctioning.

C) A 62-year-old woman was prescribed IV loop diuretics for the treatment of heart failure.

D) A 53-year-old man who received several large fluid boluses of Normal Saline.

19. Which of the following patients is at the highest risk for hypermagnesemia?

 A) A patient with alcoholism

 B) A patient taking a magnesium-containing antacids

 C) A patient suffering from Crohn’s Disease

 D) A patient with a preeclampsia receiving IV magnesium sulfate

20. Which patient is likely to present with a phosphate level of 6.0 mg/dL?

A) A patient with end-stage renal failure who was scheduled for dialysis tomorrow.
B) A patient reported drinking a 12 pack of beer daily.
C) A patient was administered an aluminum hydroxide-based antacid four times a day.
D) A patient receiving total parenteral nutrition therapy (TPN).
